# Method for selecting ornamental species for different shading intensity in urban green spaces

**DOI:** 10.3389/fpls.2023.1271341

**Published:** 2023-10-04

**Authors:** Alessandra Francini, Stefania Toscano, Antonio Ferrante, Daniela Romano

**Affiliations:** ^1^ Crop Science Research Center, Scuola Superiore Sant’Anna Pisa, Pisa, Italy; ^2^ Department of Veterinary Science, Università degli Studi di Messina, Messina, Italy; ^3^ Department of Agricultural and Environmental Sciences-Production, Landscape, Agroenergy, Università degli Studi di Milano, Milano, Italy; ^4^ Department of Agriculture, Food and Environment, Università degli Studi di Catania, Catania, Italy

**Keywords:** light compensation point, shadow projection, shade plants, sun plants, chlorophyll *a* fluorescence, photosynthesis

## Abstract

In urban areas, ornamental plants face different constraints, such as the shading of buildings and trees. Therefore, the selection of suitable species and their integration or combination with pre-existing plants is very important. Trees, shrubs, and herbaceous plant species must be distributed according to plant light requirements and shading intensity. Ornamental plants are classified into two groups based on their light intensity or shade tolerance: sun and shade species. To properly position the plants, especially in the immediate vicinity of buildings, it is necessary to study the projection of shadows during the year and the most critical periods, such as July and August. The position of ornamental species with different shading tolerances can be obtained by characterizing the leaf gas exchange for each species. Among the physiological parameters, the most important is the light compensation point, which is the light intensity corresponding to a net photosynthesis equal to zero. This means that the assimilation of carbon dioxide through photosynthesis is equal to the carbon dioxide emitted by respiration. This steady state represents the most critical condition for plants to endure the summer. The distribution of species inside a green area should be determined by considering the minimum light intensity that allows sufficient photosynthesis to compensate for the respiration rate. In this context, non-destructive leaf gas exchange, chlorophyll a fluorescence, and chlorophyll content can be useful tools for selecting suitable ornamental plants under diverse shading conditions.

## 1 Introduction

The selection of ornamental plants for building a green urban or peri-urban area is based on the aesthetic value of the plants and their ability to adapt to suboptimal conditions. Together with the experimental values, the level of maintenance requirements must also be carefully considered, particularly if the maintenance is under municipal administration. Plant maintenance includes watering, fertilization, and pruning (Aronson et al., 2017). Annual, perennial, woody shrubs, and tree species were also selected, considering their tolerance to biotic and abiotic stresses (Toscano et al., 2019). Ornamental plants are resistant to pests and diseases. This characteristic is important to reduce the need for pesticides and ensure that plants remain healthy and visually appealing. Moreover, treatment in urban areas is not permitted. The plants were selected based on their seasonal interest, including the flowering period, foliage color, and unique features, such as bark or berries. Select a combination of plants that will provide visual interest throughout the year, ensuring a vibrant and dynamic urban landscape. Urban environmental tolerance is therefore an important issue. Some species are more tolerant to urban conditions such as compacted soil, air or soil pollution, low nutrient availability, and limited space. In the selection of plants, it is also important to use non-invasive species that have the potential to escape cultivation and negatively impact native ecosystems (Leotta et al., 2023). Plants must be chosen based on their interactions with the inhabitants. In particular, plants must be safe; therefore, potential hazards associated with certain plants must be appropriately evaluated, such as thorns, toxic berries or leaves, or allergies, and plants that may pose a risk to people or pets in urban areas should be avoided. Selecting ornamental plants for urban green spaces requires careful consideration of various factors to ensure that the plants thrive and to enhance the aesthetic appeal of the area. The selection of plants can consider the most important factors that can compromise the ornamental value of the area over a long period. Under low maintenance management conditions, plants must tolerate the most frequent abiotic stresses (Francini and Sebastiani, 2019).

A combination of plants of diverse heights and those close to tall buildings should be carefully planned to avoid the negative effects of shading. Light intensity and duration are important parameters for photosynthesis and plant morphology (Cocetta et al., 2017). The lack of light can reduce the growth rate and ornamental quality. Accordingly, the combination and integration of plants must be conducted by considering the shading intensity created by buildings or other plants. Based on light requirements, ornamental species are classified as shade and sun (Bohning and Burnside, 1956). Shade plants do not require a large amount of light and grow underbrushes naturally. These plants have low light compensation points and are rich in chlorophylls. Sun plants require high light intensity and leaves with high light-compensation points. Light intensity must be sufficient to provide sufficient photosynthetic activity to accumulate sufficient sugar to satisfy basal metabolism.

The shadows in the green areas can be classified into three levels.

- Light or bright shade: The area of interest can be completely shaded for a few hours per day. The sun’s rays are blocked from buildings or vegetation for several hours during the day, but there are hours when the area is exposed to the full sun.
- Partial shade: The area is shaded for most of the day, but at least early in the morning or evening, the plants are reached by the sun’s rays.
- Complete or total shade: The area is shaded daily.

This classification system provides a simple and general guide for the selection and positioning of ornamental plants in the proposed green area.

## 2 Eco-physiological plant responses under urban environments

Light or shade intensity and temperature directly affect photosynthesis, whereas respiration is regulated only by temperature. The difference between photosynthesis and respiration represents net photosynthesis. Because photosynthesis produces sugars and respiration uses them, growth occurs when the balance between photosynthesis and respiration on a 24 h basis is positive. These physiological processes can change during different seasons, leading to the loss of ornamental quality. Therefore, they must be carefully studied to avoid positioning unsuitable plants in terms of adaptation to shade. The compensation point of different species could be a criterion for selecting ornamental plants for use in urban and peri-urban green areas. Based on these considerations, shade projections, which are the patterns of light intensity resulting from the shading of buildings and other objects, must be identified year-round, although most importantly, during spring and summer. Furthermore, temperature must also be accounted for, as net photosynthesis is temperature-dependent; increasing temperature enhances the respiration rate and reduces net photosynthesis (Evensen and Olson, 1992). Consequently, during the hottest weeks of summer, plants with a high light compensation point may produce insufficient carbohydrates for storage or growth, leading to plant death.

The relationship between the plant respiration rate and environmental temperature is well known. As the temperature increased, the respiration rate of plants also increased. This relationship can be explained by the basic principles of biochemical reactions as well as physiological and metabolic processes in plants (Atkin and Tjoelker, 2003). Plant respiration involves the breakdown of organic molecules such as sugars and carbohydrates to release energy for cellular activities. It occurs in both leaves and roots; therefore, soil temperature in urban environments is also a critical issue because of the low water availability, especially in the summer period. Several studies have investigated the relationship between plant respiration rate and temperature in urban environments (Lambrecht et al., 2016). The most evident effect was the altered phenology. Plants in urban areas can affect the timing of life cycle events including flowering, leaf emergence, and senescence. Urban plants may exhibit earlier flowering or extended growing seasons because of higher temperatures or altered light conditions in cities.

Studies performed on *Crepis sancta* (L.) Babc. plants grown in urban and rural areas showed that plants in different environments differed in phenological, morphological, and physiological traits. Plants grown in urban environments exhibit delayed flowering and senescence (Lambrecht et al., 2016). Delaying the phenological stages can be a plant strategy to accumulate sufficient resources to allow seed production for survival purposes.

## 3 Ornamental plant selection and placement

### 3.1 Photosynthesis and respiration of ornamental plants

**Figure 1:**
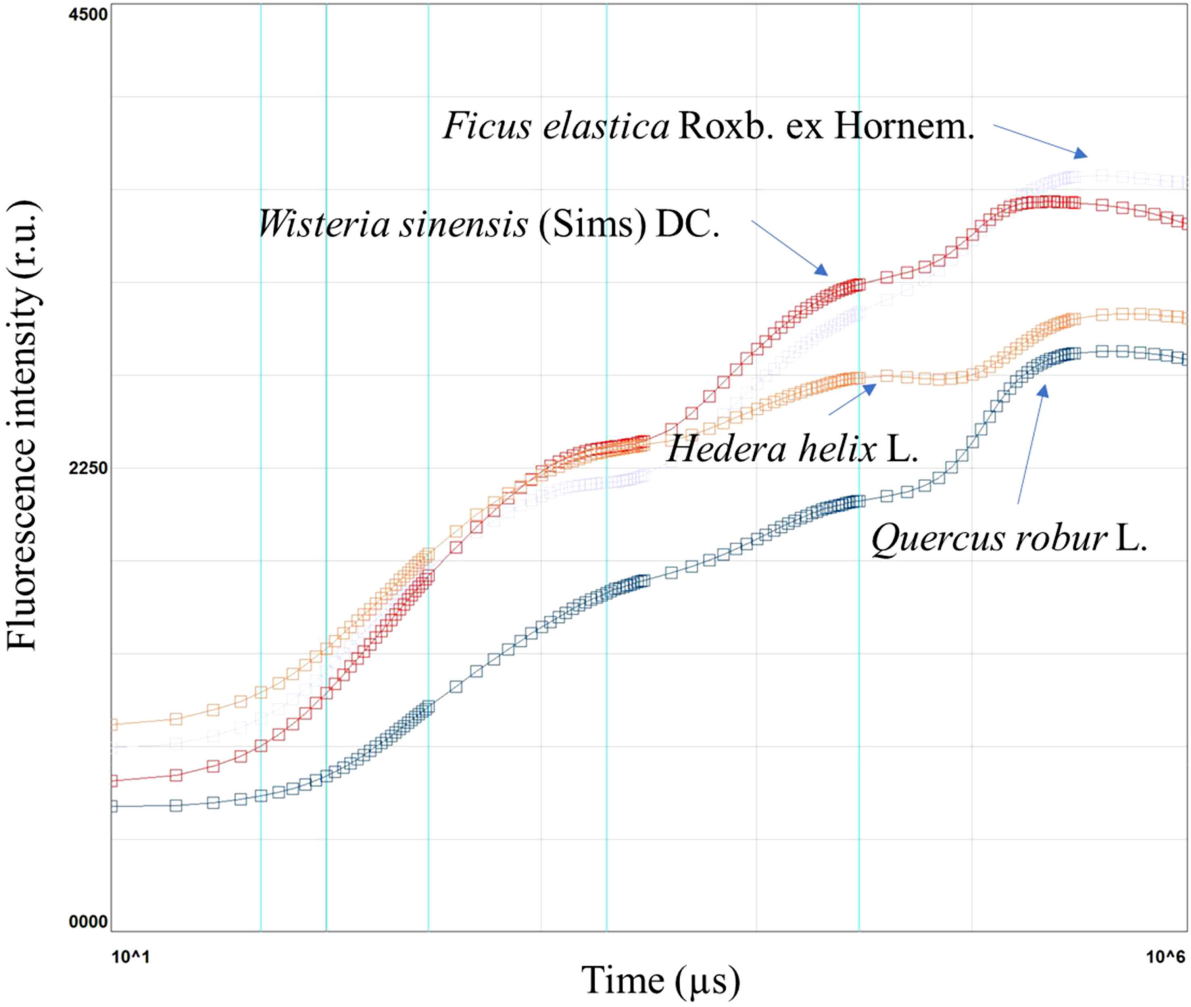
Chlorophyll *a* fluorescence induction curve in *Ficus elastica* Roxb. ex Hornem., *Wisteria sinensis* (Sims) DC., *Hedera helix* L., and *Quercus robur* L., in plants under different light conditions.

As established above, ornamental plants used in urban and peri-urban green areas should be adapted to shade and light conditions. Green shaded areas can be generated by buildings or other plants. As a general guide, shade-tolerant plants should be used in shaded areas and sun-tolerant plants in areas of full sunlight exposure. However, shade is not constant throughout the day or during the different seasons. Therefore, it is important to identify the shade intensity of trees and buildings around green areas will be planned. The correct selection of plants in the area should be carried out by matching the light compensation point of each species with the light intensity and photoperiod throughout the daily and seasonal cycles. **Figure 1** illustrates the distribution of plants with different light compensation point values.

In summer, more extensive plant canopies and higher temperatures can reduce photosynthetic activity and increase respiration, resulting in a negative net photosynthesis. Under these conditions, plants used more energy than they accumulated over 24 h. Therefore, the selection of plants should account for this, ensuring that the amount of light is sufficient to compensate for the respiration rate within a 24 h interval. The problem is less acute in spring, as plants typically do not show symptoms of shading’ however, with an increase in temperature, plants start to suffer from limited light conditions.

### 3.2 Chlorophyll *a* fluorescence measurement

Chlorophyll *a* fluorescence is a non-destructive measurement that allows the evaluation of light use efficiency, leaf functionality, and the overall leaf health status, under optimal or stress conditions. Chlorophyll *a* fluorescence is widely used to establish stressful conditions in plants. For example, it has been used to measure the chlorophyll a fluorescence in *Ficus elastica* Roxb. ex Hornem., *Wisteria sinensis* (Sims) DC., *Hedera helix* L., *Quercus robur* L.

The results showed that *F. elastica* had the highest induction curve, whereas *Q. robur* had the lowest. *W. sinensis* had a similar trend *F. elastica* and *H. helix* to *Q. robur* (**Figure 1**).

**Figure 2:**
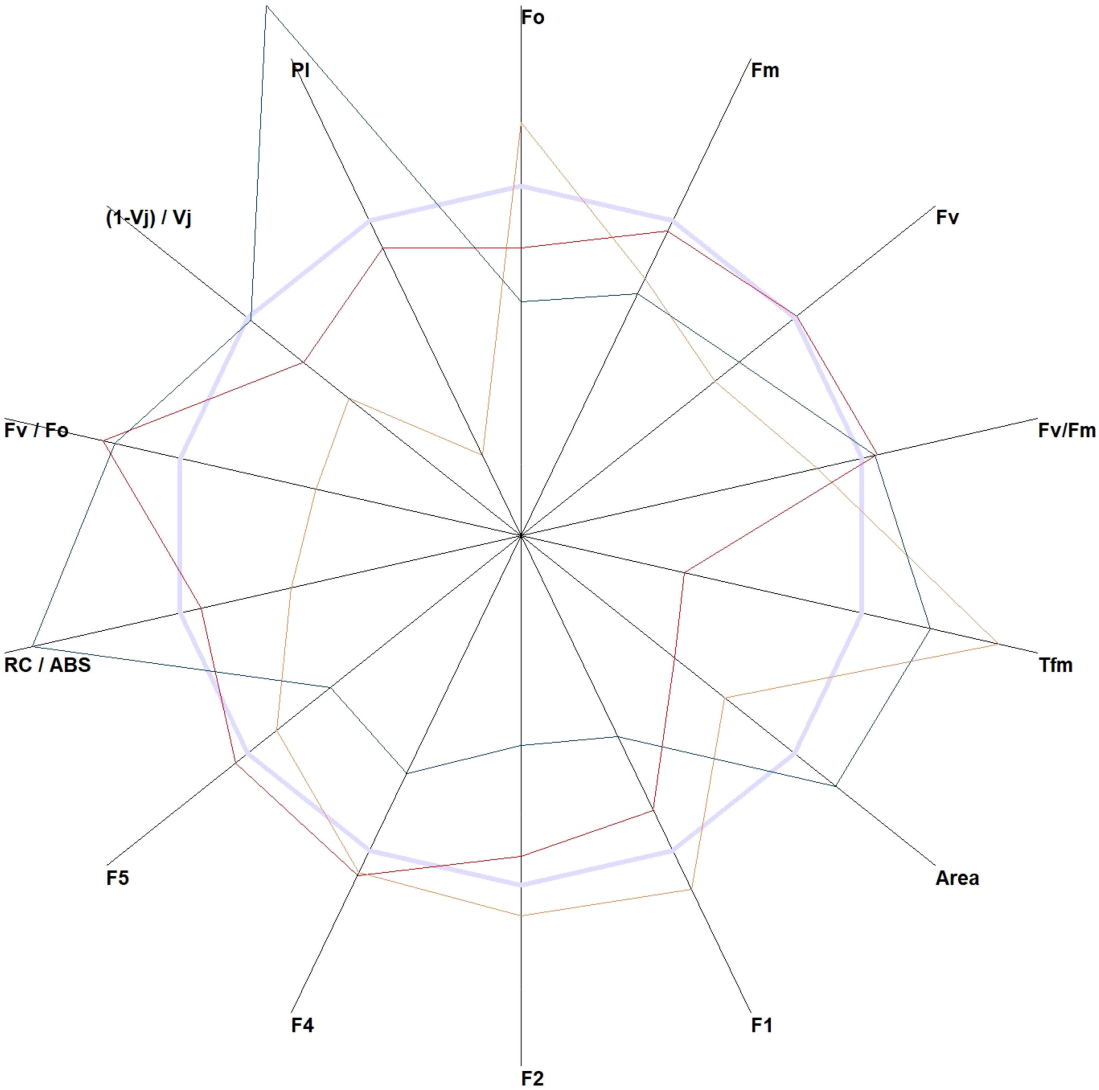
The JIP test-derived parameters were obtained from the elaboration of the chlorophyll *a* fluorescence induction curve. All parameters of *Wisteria sinensis* (Sims) DC. (red line), *Hedera helix* L. (orange line), and *Quercus robur* L. (green line) were standardized to *Ficus elastica* Roxb. ex Hornem (light blue line).

The JIP test allows the calculation of several indices from the data obtained from the data points (Tsimilli-Michael, 2020). These indices can help identify species with the highest variability. The performance index (PI), time to reach the maximal fluorescence (Tfm), and reaction centers per PSII antenna Chl a (RC/ABS) are the parameters that could greatly show the differences in plants under different light conditions (**Figure 2**). An expanded list of the JIP indices is provided in **Supplementary Table 1**.

### 3.3 Adaptation of plants to light conditions

Plants can adapt to suboptimal conditions. In light compensation points of ornamental plants under low- or high-light conditions are reported in **Table 1**. Plants can alter their light compensation points by adapting to various environments. For example, *Pseudomonas scutellarioides* R. Br. reported an increase in the light compensation points in plants grown under high-pressure sodium lamps (HPS) or light-emitting diodes (LEDs) (Domurath et al., 2012). Lower compensation points are required for plants used in indoor environments, such as *Philodendron erubescens* K. Koch & Augustin and *Dracaena surculosa* Lindl. (Tan et al., 2017), whereas an analogous experiment simulating indoor conditions by lowering the light intensity resulted in reduced light compensation points in *Leea coccinea* Planch and *L. rubra* Blume ex Spreng. (Sarracino et al., 1992). Different light compensation points can also be observed in the same plant under different light-exposure conditions (Zhao et al., 2012). Leaves of *Paeonia lactiflora* Pall. exposed to the sun showed double the light compensation point compared with those under shade. This information can be useful when selecting plants for use in shaded locations, as adaptation may be used to lower the light composition of a species at the nursery level by applying different shading nets.

**Table 1:** Light compensation points (LCPs) of different species and light intensities are recommended in the area during the day of the most critical season.

Species	Light compensation point (LCPs) (μmol m^−2^ s^−1^)	Light intensity recommended (μmol m^−2^ s^−1^) × 12 h	Reference
*Aeschynanthus longicaulis* Wall. ex R.Br.	9.1–21.9	>11–24	Li et al. (2014)
*Chlorophytum comosum* (Thunb.) Jacques	10	>12	Torpy et al. (2017)
*Epipremnum aureum* (Linden ex André) G.S.Bunting	10	>12	Torpy et al. (2017)
*Ficus benjamina* L.	5.9	>6	Scuderi et al. (2012)
*Gibasis* Raf. spp.	10	>12	Torpy et al. (2017)
*Heuchera americana* L.	3.47–4.96	>4.2–5.9	Garland et al. (2012)
*Leea coccinea* Planch.	52–88	>63–106	Sarracino et al. (1992)
*Leea rubra* Blume ex Spreng.	67–84	>81–101	Sarracino et al. (1992)
*Neomarica* Sprague spp.	15	>18	Torpy et al. (2017)
*Paeonia lactiflora* Pall.	7–13	>8.4–16	Zhao et al. (2012)
*Passiflora morifolia* Mast.	36–39	>43–47	Pires et al. (2011)
*Passiflora palmeri* var. *sublanceolata* Killip	16–21	>19–25	Pires et al. (2011)
*Passiflora suberosa* subsp*. litoralis* (Kunth) Port.-Utl. ex M.A.M.Azevedo, Baumgratz & Gonç.-Estev.	27–32	>32–38	Pires et al. (2011)
*Physocarpus amurensis* (Maxim.) Maxim.	20	>24	Zhang et al. (2016)
*Physocarpus opulifolius* (L.) Maxim.	20	>24	Zhang et al. (2016)
*Peperomia* Ruiz & Pav. spp.	13	>16	Torpy et al. (2017)
*Philodendron erubescens* K.Koch & Augustin or *Dracaena surculosa* Lindl.	6–12	>7.2–14.4	Tan et al. (2017)
*Philodendron xanadu* Croat, Mayo & J.Boos	14	>17	Torpy et al. (2017)
*Plectranthus scutellarioides* R.Br.	15	>18	Domurath et al. (2012)

The recommended light intensity should be 20% higher than the LCP within the range of 12 h.

*Aeschynanthus longicaulis* Wall. ex R.Br., the morphological and photosynthetic responses were measured under high or low irradiance (Li et al., 2014). Under high irradiance, the photosynthetic photon flux density (PPFD) was maintained under 650 µmol·m^−2^·s^−1^ by internal and external shading of the greenhouse. The light intensity of the low irradiance treatment was maintained under 150 µmol·m^−2^·s^−1^ by extra shading on the bench (Li et al., 2014). The LCP of *Athyrium pachyphlebium* C. Chr. at four different shade ranges was 813 μmol m^–2^ s^–1^ to 886 μmol m^–2^ s^–1^, 576 μmol m^–2^ s^–1^ to 633 μmol m^–2^ s^–1^, 335 μmol m^–2^ s^–1^ to 402 μmol m^–2^ s^–1^, 134 μmol m^–2^ s^–1^ to 175 μmol m^–2^ s^–1^ and 1,846 μmol m^–2^ s^–1^ to 1,914 μmol m^–2^ s^–1^. The LCP under the highest light regime was 33 μmol m^–2^ s^–1^ to 16 μmol m^–2^ s^–1^ (Huang et al., 2011). Similar study was performed on *Passiflora morifolia* Mast., *P. suberosa* subsp*. litoralis* (Kunth) Port.-Utl. ex M.A.M.Azevedo, Baumgratz and Gonç.-Estev., and *P. palmeri* var. *sublanceolata* Killip (Pires et al., 2011). These ornamental plants showed different LCPs ranging from 36 μmol m^–2^ s^–1^ to 21 μmol m^–2^ s^–1^ under reduced light intensities of 25%, 50%, and 75% solar radiation for evaluating the adaptation ability (Pires et al., 2011). Artificial shade was provided using different shading nylon nets, fixed in wooden frames with dimensions of 5 m × 5 m × 2 m, under field conditions, which allowed a 25% reduction (1,000 μmol m^–2^ s^–1^–1,400 μmol m^–2^ s^–1^, min–max), 50% reduction (600 μmol m^–2^ s^–1^ –900 μmol m^–2^ s^–1^), and 75% reduction (200 μmol m^–2^ s^–1^ –400 μmol m^–2^ s^–1^) of solar radiation, along with a control treatment under full sunlight (1,500 μmol m^–2^ s^–1^–1,900 μmol m^–2^ s^–1^). The reduced light intensity by 75% allowed the LCP reduction in all three species and the highest plasticity was observed in *P. suberosa* subsp*. litoralis*, and *P. palmeri* var. *sublanceolata* (Pires et al., 2011). The shading effect of LCP and plant growth were studied in *Ficus benjamina* L. grown under five shading conditions during two growing periods (Scuderi et al., 2012). The control (0% shading) and 80% shading levels had light intensities ranging from 880 μmol·m^−2^·s^−1^ to 142 μmol·m^−2^·s^−1^ in the first growing period and from 1,531 μmol·m^−2^·s^−1^ to 173 μmol·m^−2^·s^−1^ in the second growing period. Under these shading regimes, the LCP of *F. benjamina* was 17 μmol m^–2^ s^–1^ and 5.9 μmol m^–2^ s^–1^ (Scuderi et al., 2012).

Two species, *Physocarpus amurensis* (Maxim.) Maxim. and *P. opulifolius* (L.) Maxim. were exposed to low (100 μmol m^–2^ s^–1^) or high light intensity (1,000 μmol m^–2^ s^–1^ –1,500 μmol m^–2^ s^–1^), and their LCP respected the two conditions. The LCP values were 26 μmol m^–2^ s^–1^ and 28 μmol m^–2^ s^–1^ for *P. amurensis* and *P. opulifolius*, respectively, under high-irradiance conditions. At low irradiance, both species have an LCP of 20 μmol m^–2^ s^–1^ (Zhang et al., 2016).

Shade and sun exposure conditions were studied in *Calophyllum inophyllum* L. (Clusiaceae), *Inga spectabilis* (Vahl) Willd. (Fabaceae), and *Ormosia macrocalyx* Ducke (Fabaceae). *C. inophyllum* and *I. spectabilis* under shade showed a LCP three times lower than plants exposed to sun. *O. macrocalyx* only halved the LCP when exposed to shade (Slot et al., 2019).

Several plants have been adapted to indoor conditions to evaluate the ability of some species to remove carbon dioxide from the environment (Treesubsuntorn and Thiravetyan, 2018). The LCP of these species ranges from 10 µmol·m^−2^·s^−1^ to 15 µmol·m^−2^·s^−1^ (Torpy et al., 2017).

**Figure 3:**
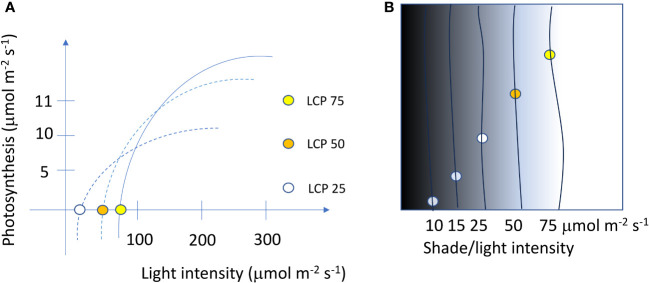
Light saturation curves and light compensation points (LCPs) of three different ornamental species **(A)** and the positioning of plants in different shade or light intensities that match the requirement of LCP on a 24 h basis **(B)**.

Based on experimental or literature information (Ignatieva, 2021), plants can be planted in various green areas. If we consider plants with LCP 25 µmol·m^−2^·s^−1^, 50 µmol·m^−2^·s^−1^, and 75 µmol·m^−2^·s^−1^ (**Figure 3A**) the possible planting position/zone in the area mapped for the shade/light intensity are reported in **Figure 3B**.

Considering the information reported in **Table 1** in the shade area with light intensity below 10 µmol·m^−2^·s^−1^ can be planted the *Heuchera americana* L. In areas comprised between 10 and 15 can be planted species such as *Chlorophytum comosum* (Thunb.) Jacques, *Epipremnum aureum* (Linden ex André) G.S.Bunting, *Ficus benjamina* L., and *Gibasis* Raf. spp. In areas with higher light intensities, the number of species that can be used greatly increased (**Table 1**). Of course, species must be selected based on the temperature of the geographical area in winter and these temperatures must be compatible with species tolerance.

## 4 Materials and methods

### 4.1 Protocol and procedures for the identification of LCP and chlorophyll *a* fluorescence measurements

Ornamental plants must be maintained under optimal nutrition and water conditions. The light saturation curve and light compensation point should be measured under specific light intensities that should be set from 1,000 µmol m^−2^ s^−1^ to 1,600 µmol m^−2^ s^−1^ for sun plants, 100 µmol m^−2^ s^−1^–200 µmol m^−2^ s^−1^ for shade plants and of 500 µmol m^−2^ s^−1^–700 µmol m^−2^ s^−1^ for intermediate conditions. The plasticity of plants to adapt to low light regimes must be evaluated by transferring plants under shading and reducing the light intensity using nets with a reduction of 25%, 50%, or 75% solar radiation. Measurements should be carried out after one week of adaptation.

### 4.2 Light saturation curve and light compensation point determinations

The light saturation curve and light compensation point of a plant can be measured using a leaf gas exchange analyzer. This instrument allows for the identification of the light compensation point and light intensity that allows the achievement of the highest net photosynthesis. The light saturation curve can be determined by measuring the CO_2_ assimilation by decreasing the light intensity from 1,600 μmol m^−2^ s^−1^ to 0 μmol m^−2^ s^−1^. The CO_2_ concentration should be at ambient level (400 µL L^−1^). Readings were taken between 9:30 A.M. and 11:30 A.M. from the fully expanded leaves of the three plants.

### 4.3 Chlorophyll *a* fluorescence determination

**Figure 4:**
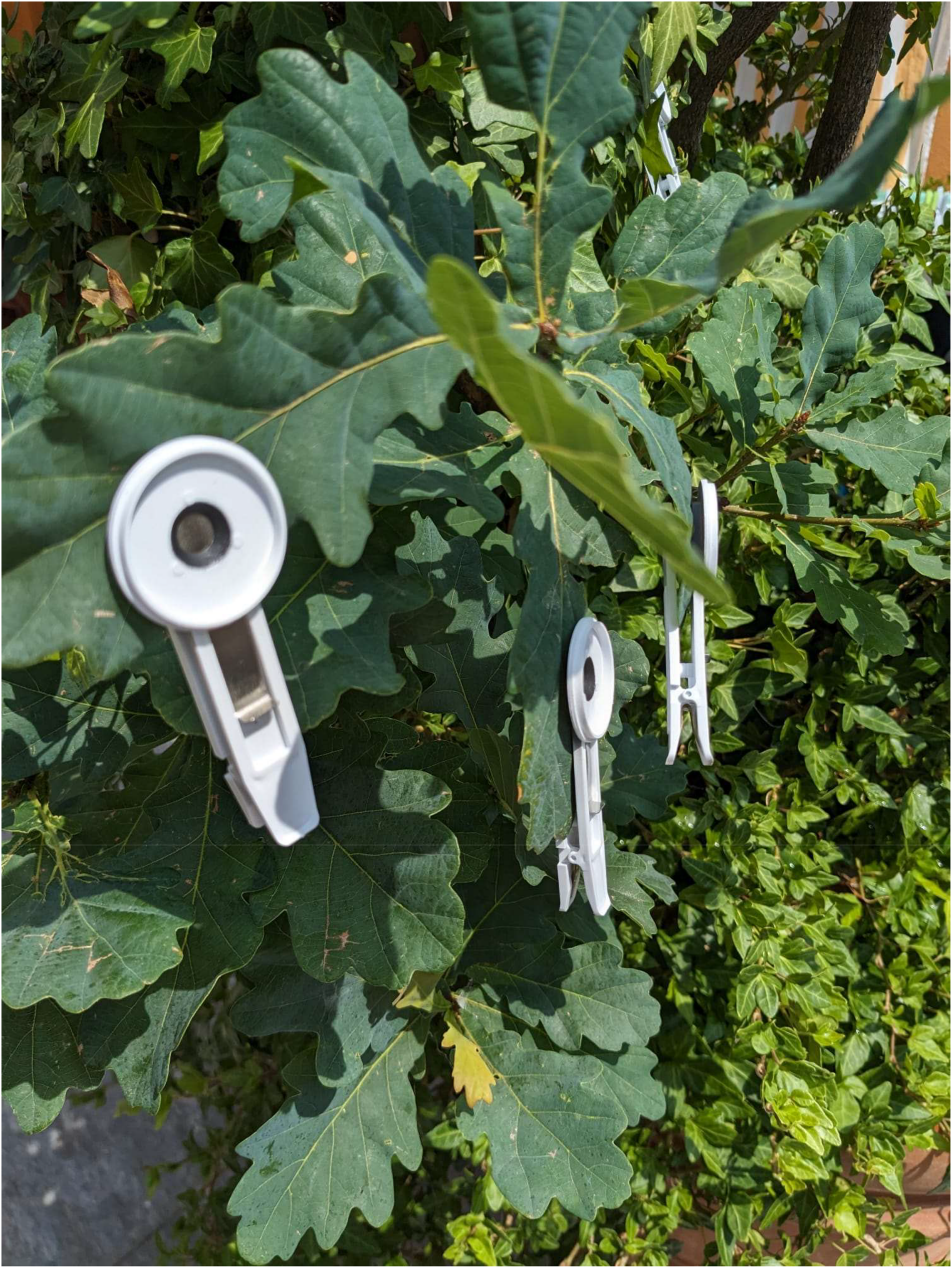
Leaf clip used for dark incubation of leaf area. The leaf clip should be placed on fully expanded and mature leaves. The incubation period should be at least 40 min to allow complete oxidation of PSII.

Chlorophyll *a* fluorescence can be measured using a portable fluorimeter that allows the determination of plant health status, light-use efficiency, and plant adaptability to different light conditions. The chlorophyll *a* fluorescence induction curve was measured using a Handy Plant Efficiency Analyzer (PEA, Hansatech, UK). Chlorophyll *a* fluorescence can be measured in leaves that must be dark-adapted for 30 min by using a leaf clip (4 mm diameter). A rapid pulse of high light intensity of 3,300 μmol m^−2^ s^−1^ (600 W m^−2^) was emitted on the leaf area shaded by the leaf clip (**Figure 4**). The sensor recorded the leaf fluorescence. Chlorophyll a fluorescence parameters, such as Fo (minimal fluorescence), Fm (maximum fluorescence), Fv (variable fluorescence), and Fv/F_M_ ratio (maximum quantum efficiency of photosystem II), were automatically calculated. JIP analysis can be performed to determine several indices related to light use efficiency and plant adaptation (**Supplementary Table 1**).

### 4.4 Distribution of plants in the green area

Based on the data collected regarding the light compensation point, it was possible to distribute the plants in the green areas. Light availability at the planting position must be higher than the light intensity of the light compensation point under the worst conditions, such as in summer with high temperatures.

## 5 Conclusion

The success of building a green area depends on many environmental factors including soil properties, temperature, relative humidity, and light or shade intensity. In particular, the low-light conditions that plants can be exposed to during the summer season must be considered. Correct ornamental plant species selection should be performed considering the light compensation point of the species and the shade intensity in the green area generated by tall trees or buildings.

## Data availability statement

The original contributions presented in the study are included in the article/**Supplementary material**, further inquiries can be directed to the corresponding author/s.

## Author contributions

AlF: Writing – original draft. ST: Writing – review & editing. AnF: Conceptualization, Writing – original draft, Writing – review & editing. DR: Supervision, Writing – review & editing.
