# Supplementary material for: Method for selecting ornamental species for different shading intensity in urban green spaces
Source: Front Plant Sci. 2023 Oct 4;14:1271341. doi: 10.3389/fpls.2023.1271341 (PMC10582628; doi:10.3389/fpls.2023.1271341)
Supplement: Supplementary file 1 [file Table_1.pdf]

Supplementary Material

Method for selection ornamental species for different shading intensity in urban green spaces

Alessandra Francini, Stefania Toscano*, Antonio Ferrante, Daniela Romano

*** Correspondence:** Corresponding Author: stefania.toscano@unime.it

Supplementary Table 1. Glossary of terms and formulae used by JIP-test for the analysis of the transient fluorescence O-J-I-P

1 Data extracted from the recorded chlorophyll a fluorescence transient OJIP

F_t, fluorescence at time t after onset of actinic illumination

$F_{20\mu s}$ or $F_{50\mu s}$, minimal reliable recorded fluorescence, at 20 or 50 µs, that is commonly named Fo

$F_{100\mu s}$, fluorescence at 100 µs

$F_{300\mu s}$, fluorescence at 300 µs

F_J = F2ms, fluorescence at the J-step (2 ms) of OJIP

F_I = F30ms, fluorescence at the I-step (30 ms) of OJIP

$F_p = F_M$, maximal fluorescence at the peak P of OJIP

t_{F_M}, time to reach the maximal fluorescence F_M

Area, total complementary area between fluorescence curve and $F= F_M$

2 Fluorescence parameters derived from the extracted data

Fo = $F_{20\mu s}$ or $F_{50\mu s}$, minimal reliable recorded fluorescence, when all PSII Reaction Centres (RCs) are open (at t=0)

$F_M=F_P$, maximal fluorescence, when all PSII RCs are closed

$F_v=F_t$-Fo, variable fluorescence at time t

$F_V=F_M$-Fo, maximal variable fluorescence

$V_t=(F_t-Fo)/(F_M-Fo)$, relative variable fluorescence at time t

V_J=(F_J-Fo)/(F_M-Fo), relative variable fluorescence at the J-Step

W_t=(F_t-Fo)/(F_J-Fo), W at 100 µs of a simulated exponential fluorescence F_v to the amplitude F_J-Fo

$W_{E,\ 100\ \mu s}$ = 1-(1-$W_{300\ \mu s}$), W at 100 µs of a simulated exponential fluorescence transient corresponding to the sample in the absence of grouping (i.e., no connectivity between PSII units)

Mo= (ΔV/Δt)o = 4 ($F_{300\ \mu s}$ -Fo)/(F_M-Fo), approximated initial slope of transient fluorescence V=f(t)

S_m = (Area)/(F_M-Fo), normalized total complementary area above the OJIP transient (multiple turnover Q_A reduction events)

S_s=V_J/Mo, normalized total complementary area corresponding only to the O-J phase (single turnover Q_A reduction events)

N = S_m/S_s =S_m Mo (1/V_J), turnover number: number of Q_A reduction events between time 0 and t_{F_M}

V_m = 1-(S_m/t_{F_M}), average relative variable fluorescence from time 0 to t_{F_M}

3 Specific energy fluxes

ABS/RC = Mo (1/V_J) (1/φ_{Po}), absorption flux per RC

TRo/RC=Mo (1/V_J), trapped energy flux per RC

ETo/RC = Mo (1/VJ) ψ_o, electron transport flux per RC

DI_o/RC = (ABS/RC) – (TRo/RC), dissipated energy flux per RC

4 Yields or flux ratios

φ_{Po} = TRo/ABS = [1-(F_v/FM)], maximum quantum yield of primary photochemistry (at t = 0)

ψ_v = ETo/TRo = (1-V_J), probability (at t = 0) that a trapped exciton moves an electron into the electron transport chain beyond Q^-_A

φ_{Eo} = Eto/ABS = [1-(Fo/F_M) ψ_o, quantum yield of electron transport (at t = 0)

φ_{Do} = 1- φ_{Po} = (Fo/F_M), quantum yield (at t = 0) of energy dissipation

φ_{Pv} = φ_{Po} (1-Vv) = φ_{Po} (S_m/t_{F_M}), average (from time 0 to t_{F_M}), quantum yield of primary photochemistry.
